# Comprehensive analysis of potential cellular communication networks in advanced osteosarcoma using single-cell RNA sequencing data

**DOI:** 10.3389/fgene.2022.1013737

**Published:** 2022-10-11

**Authors:** Ning Xu, Xiaojing Wang, Lili Wang, Yuan Song, Xianyou Zheng, Hai Hu

**Affiliations:** ^1^ Departments of Orthopedics, Shanghai Eighth People’s Hospital, Shanghai, China; ^2^ Department of Neurology, The First Affiliated Hospital of Anhui Medical University, Hefei, China; ^3^ Departments of Orthopedics, Sixth People’s Hospital Affiliated to Shanghai Jiao Tong University, Shanghai, China

**Keywords:** osteosarcoma, cell types, cellular communication networks, regulon activity, ScRNA-seq

## Abstract

Osteosarcoma (OS) is a common bone cancer in children and adolescents, and metastasis and recurrence are the major causes of poor treatment outcomes. A better understanding of the tumor microenvironment is required to develop an effective treatment for OS. In this paper, a single-cell RNA sequencing dataset was taken to a systematic genetic analysis, and potential signaling pathways linked with osteosarcoma development were explored. Our findings revealed 25 clusters across 11 osteosarcoma tissues, with 11 cell types including “Chondroblastic cells”, “Osteoblastic cells”, “Myeloid cells”, “Pericytes”, “Fibroblasts”, “Proliferating osteoblastic cells”, “Osteoclasts”, “TILs”, “Endothelial cells”, “Mesenchymal stem cells”, and “Myoblasts”. The results of Cell communication analysis showed 17 potential cellular communication networks including “COLLAGEN signaling pathway network”, “CD99 signaling pathway network”, “PTN signaling pathway network”, “MIF signaling pathway network”, “SPP1 signaling pathway network”, “FN1 signaling pathway network”, “LAMININ signaling pathway network”, “FGF signaling pathway network”, “VEGF signaling pathway network”, “GALECTIN signaling pathway network”, “PERIOSTIN signaling pathway network”, “VISFATIN signaling pathway network”, “ITGB2 signaling pathway network”, “NOTCH signaling pathway network”, “IGF signaling pathway network”, “VWF signaling pathway network”, “PDGF signaling pathway network”. This research may provide novel insights into the pathophysiology of OS’s molecular processes.

## Introduction

Osteosarcoma (OS) is a highly malignant solid bone tumor characterized by malignant mesenchymal cells producing pathological osteoid and/or bony matrix; it accounts for roughly 60% of all pediatric malignancies (Bousquet et al., 2016; Guo et al., 2022; Ho et al., 2017), and the incidence of OS in the overall population is two to three million per year (Shao et al., 2022). Clinical signs of OS affect the proximal tibia, proximal humerus, and distal femur, and consist predominantly of local discomfort, edema, and reduced joint movement (Rothzerg et al., 2021). Currently, this cancer is treated with surgical excision and chemotherapy with many agents. Unfortunately, the 5-years overall survival rate for osteosarcoma patients was just approximately 60% among patients with localized osteosarcoma but is only 20% among patients presenting with metastases or recurrent disease (Meltzer and Helman, 2021). The pathophysiology of OS is characterized by the substantial infiltration of complex cells, including malignant mesenchymal stem cells, proliferating osteoblastic cells, osteoblastic cells, immunological cells, and vascular networks, indicating the existence of a highly complex tumor microenvironment (TME) (Kansara et al., 2014). Nonetheless, the potential cellular communication networks of these cells are still not fully elucidated.

To understand cancer biology and immunology and to get the most out of tumor immunotherapy, it is important to figure out how this ecosystem’s cells work together and how they might talk to each other. The ultimate unit of biological activity is a single cell, where genetic processes interact with the cellular environment to determine the development and function of complex structures including tissues and organs. Understanding the biology of virtually all living phenomena in normal and disease states necessitates dissecting and characterization of their composition and characterization, as well as evaluating their interactions, dynamics, and function at the single-cell level (Ren et al., 2018). Technically, however, previous genomic, transcriptomic, and proteomic cancer investigations have been unable to comprehensively elaborate on TME due to its complexity.(Liu et al., 2021). The emergence of new technologies based on single-cell sequencing has enabled unparalleled resolution and scale in capturing diverse tumor stages and understanding tumor heterogeneity (Vegliante et al., 2022). Rapid advancements in single-cell technology provide us with a potent approach to examine the multiple allosteric states and potential cellular communication networks of the TME at the single cell level.

This study employed scRNA-seq to investigate potential cellular communication networks in the OS’s TME, as well as trajectory analysis and transcription factor enrichment analysis among mesenchymal stem cells, proliferating osteoblastic cells, and osteoblastic cells.

## Materials and methods

### Data source collection and processing

The 11 OS samples with scRNA-seq data based on the 10X Genomics platform were downloaded from GSE152048 via the Gene Expression Omnibus database (https://www.ncbi.nlm.nih.gov/geo/). The Seurat package (v4.1.1) was used to load the 10X genomics data for each individual sample into R software (v4.1.3). We excluded cells with identified genes <300 or a percentage of mitochondrial genes over 10% of total expressed genes. We eliminated low-quality cells with ≥7,500 detected genes, as well as genes detected in fewer than three cells. Furthermore, using the DoubletFinder package (v2.0.3), we eliminated any doublets that might have happened during encapsulation or as random pairings of cells that were not separated during sample preparation.

This research did not need ethical approval for our work because we used data from a publicly accessible database.

### Identification of cell types

For each cell, gene expression was expressed as a fraction of the gene multiplied by 10,000, The log (x+1) method was used to perform natural log transformation. We identified, and scaled the top 2000 highly variable genes (HVGs) from the normalized expression matrix before doing principal component analysis (PCA) on these HVGs. Based on the top 50 PCA components identified, the batch effects were removed using the R Harmony package (version 1.0) (Zhou et al., 2020). On the basis of harmony-corrected data, k-nearest neighbors were estimated, and a shared nearest neighbor (SNN) graph was formed. The modular function was then adjusted to achieve cluster recognition based on the clustering algorithm. On the 2D map generated with the t-distributed stochastic neighbor embedding (tSNE) or uniform manifold approximation and projection for dimension reduction (UMAP) approach, the identified clusters were displayed.

Using the “FindAllMarkers” function, each cluster’s marker genes were identified according to the following criteria: logfc. threshold = 0.25, min. pct = 0.25, and min. diff.pct = 0.25. Using the “DotPlot” tool in Seurat, the expression pattern of each marker gene across clusters was shown. The cell groupings were annotated based on the DEGs and well-known cellular markers described in the scientific literature (Zhou et al., 2020).

### Pseudotemporal ordering of single cells

The Monocle package (v2.22.0) was used to produce the single-cell pseudotime trajectories. Using pseudotemporal profiling of scRNA-seq data, Monocle aims to decipher cellular changes during differentiation. After inputting the scale of raw UMI counts into the “newCellDataSet” function with its clustering information, it was computed into a lower dimensional space using the discriminative dimensionality reduction with trees (DDRTree) method, a more recent manifold learning algorithm. Mesenchymal stem cells, proliferating osteoblastic cells, and osteoblastic cells were then ordered according to pseudotime. The plot pseudotime heatmap was used to compute and illustrate the genes whose expression varied in tandem with pseudotime.

### Cell-cell communication

The CellChat package (version 1.4.0) predicted cell–cell communication across all cell types based on single-cell RNA sequencing data (Jin et al., 2021). Only the ligand–receptor interaction with a *p*-value 0.05 was utilized to predict cell–cell interaction in the various cell types.

### SCENIC analysis

SCENIC is a technique that uses scRNA-seq data to rebuild gene regulation networks while also recognizing stable cell states. Transcription factor enrichment and regulon activity were assessed using SCENIC package (version 1.3.1) is introduced (Aibar et al., 2017). Based on co-expression and DNA motif analysis, the gene regulatory network was created, and the network activity in each cell was assessed to determine the cell state. For transcription factor regulatory network development, two gene-motif rankings (10 kb around the transcription start site or 500 bp upstream and 100 bp downstream of the transcription start site) were used as a guide to set the search space around the transcription start site. The gene-motif rankings for humans are obtained from https://resources.aertslab.org/cistarget/. The database used was Hallmark Gene Set from Molecular Signatures database (MsigDB) (Liberzon et al., 2015). In addition, Gene regulation was constructed using the R package GENIE3 (version 1.16.0), RcisTarget (version 1.14.0) and AUCell (version 1.16.0).

## Results

### Quality control and removal of batch effect

**FIGURE 1:**
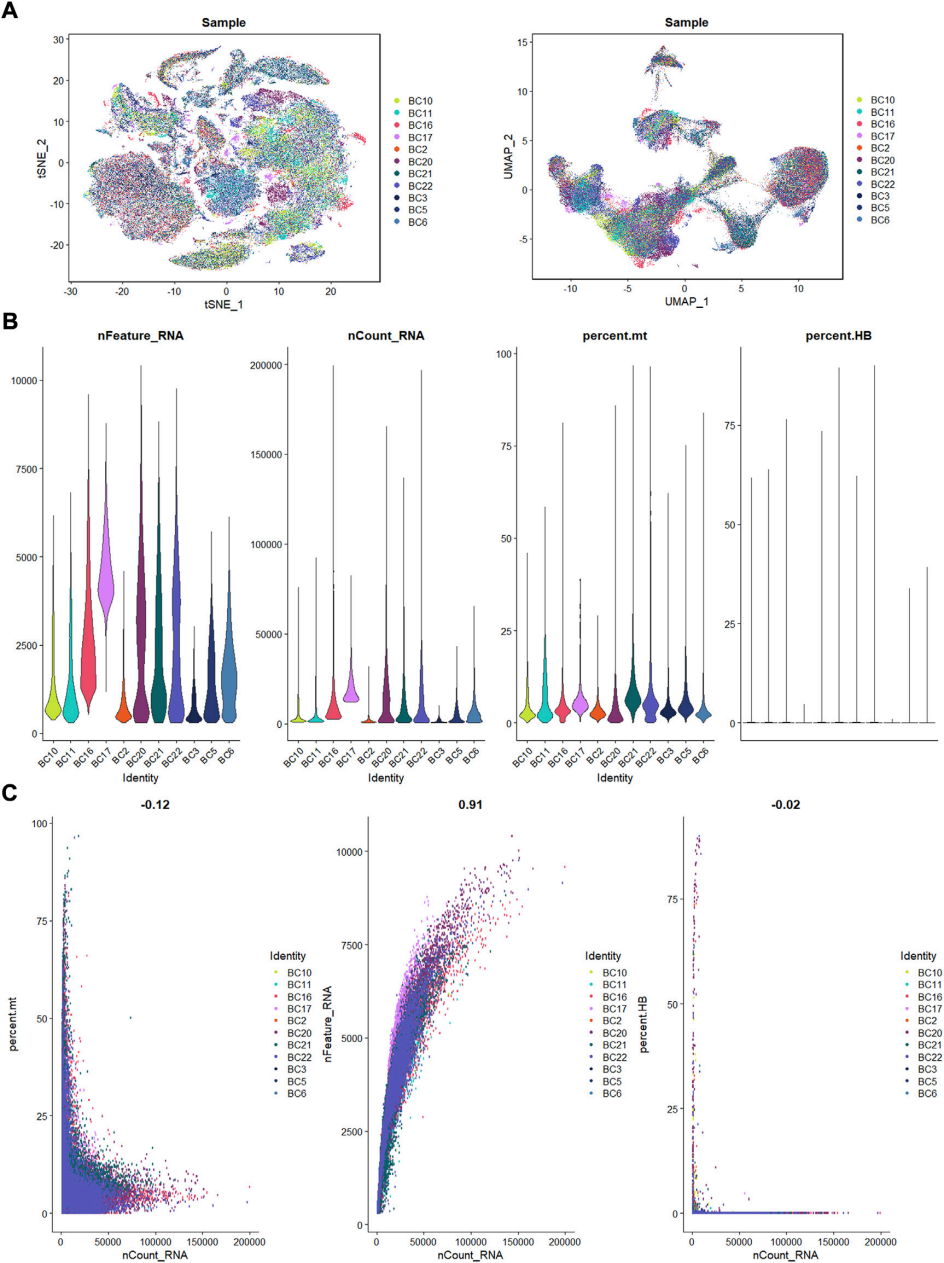
The process of quality control. **(A)**: t-SNE and UMAP plots after harmony. **(B)**: violin plots of feature, count, percent. mt, and percent.HB. **(C)**: correlation plots for count and feature, percent. mt, percent. HB.

Eleven OS patients with scRNA-seq data enrolled in this research. Using the R Harmony package (version 1.0), batch effects between samples were eliminated based on the top 50 PCA components. After removal of batch effect, we used the t-SNE and UMAP techniques to decrease dimensionality, and then plotted the result as a 2D scatter plot (Figure 1A). In the process of quality control, we eliminated cells with fewer than 300 identified genes or a proportion of mitochondrial genes exceeding 10% of all expressed genes (Figure 1B). Dot plot of data quality control in scRNA-seq data were shown in Figure 1C.

### Identification of 25 cell clusters in osteosarcoma microenvironment using scRNA-seq data reveals high cell heterogeneity

**FIGURE 2:**
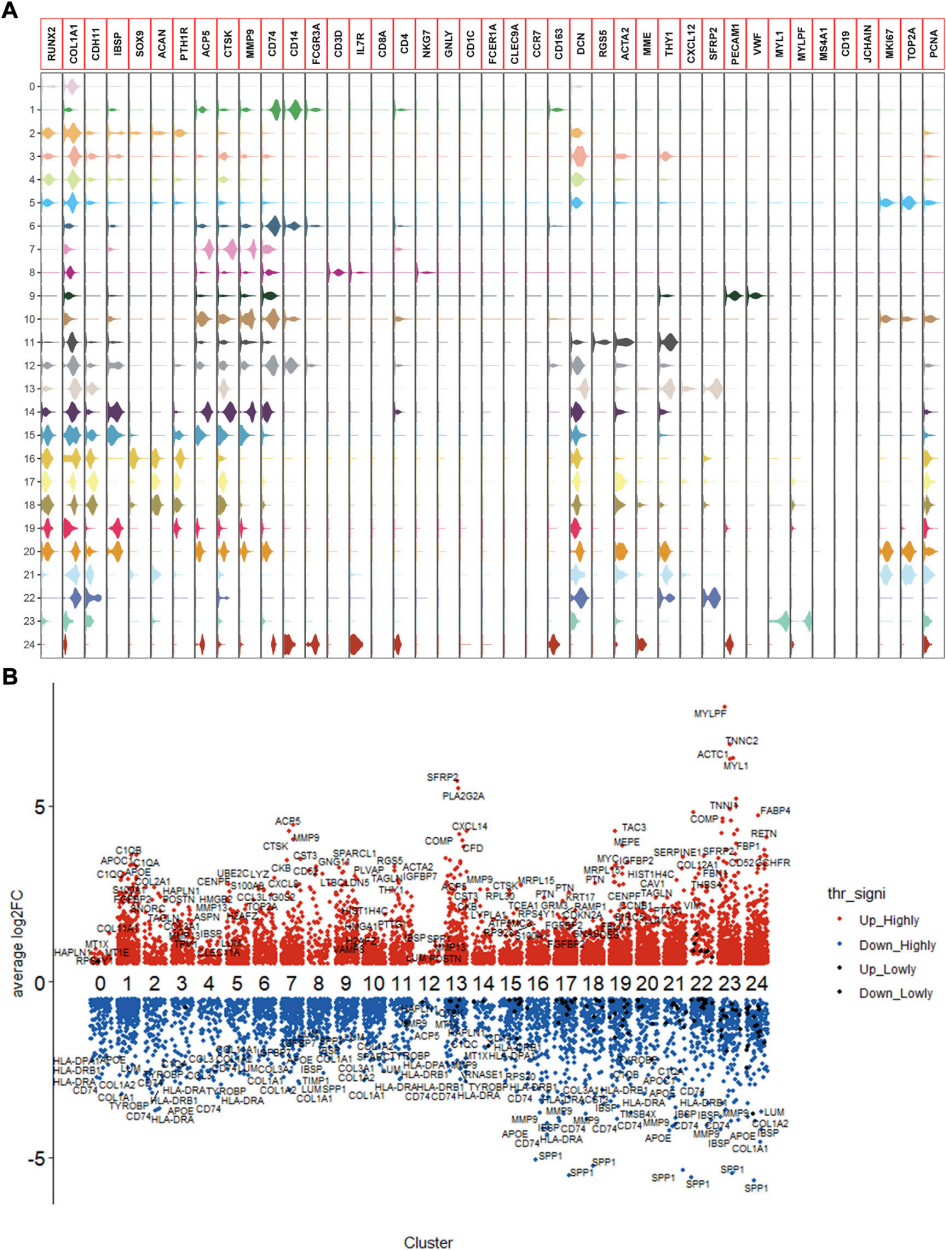
Expression of marker genes in the OS. **(A)**: violin plot of marker genes. **(B)**: columnar scatter plot of DEGs.

**FIGURE 3:**
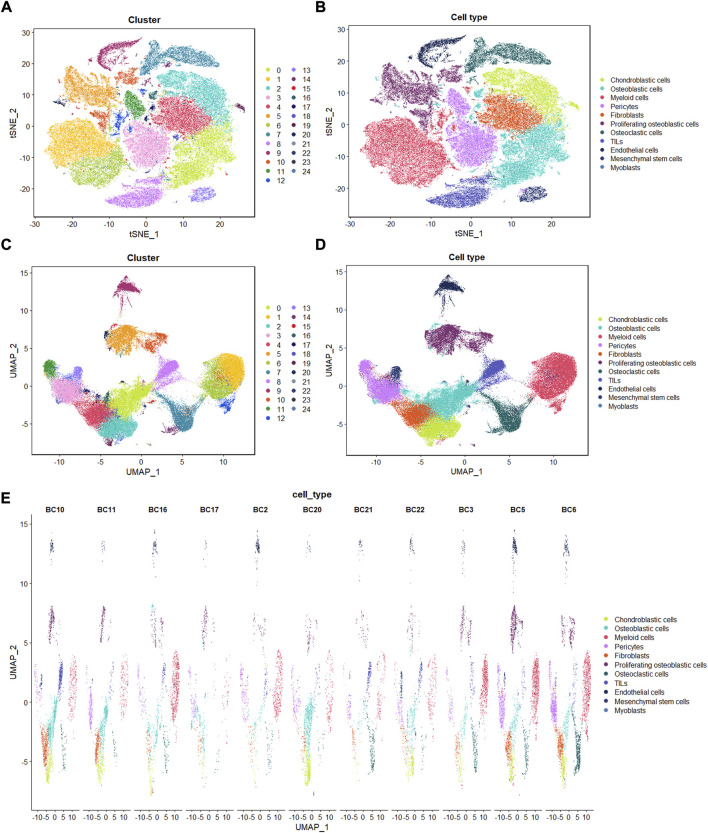
Single-cell transcriptomic analysis of OS lesions. **(A,C)**: t-SNE and UMAP analysis showing the results of descending clustering. **(B,D,E)**: t-SNE and UMAP analysis showing the results of annotation of cell subpopulations in cells of OS tissues.

Following the quality control standard, 110,042 cells were finally included in our analysis. These cells were clustered into 25 primary cell clusters (Figures 2A,B; Figures 3A,C). A value of adjusted *p* value <0.01 is displayed in red, whereas a value of adjusted *p* value ≥0.01 is displayed in black (Figure 2B). Analysis of differential gene expression revealing up- and down-regulated genes in all clusters. The cluster-specific markers were utilized to label cell types (Figures 3B,D,E): chondroblastic cells (Sox9, Acan, Pth1r), osteoblastic cells (Runx2, Col1a1, Cdh11, Ibsp), myeloid cells (Cd74, Cd14, Fcgr3a), pericytes (Rgs5, Acta2), fibroblasts (Dcn, Col1a1), proliferating osteoblastic cells (Mki67, Top2a, Pcna), osteoclasts (ACP5, Ctsk, Mmp9), TILs (IL7R, CD3D, NKG7), endothelial cells (Pecam1, Vwf), mesenchymal stem cells (Mme, Thy1, Cxcl12, Sfrp2), and myoblasts (Myl1, Mylpf).

### Potential cellular communication networks in the osteosarcoma microenvironment

**FIGURE 4:**
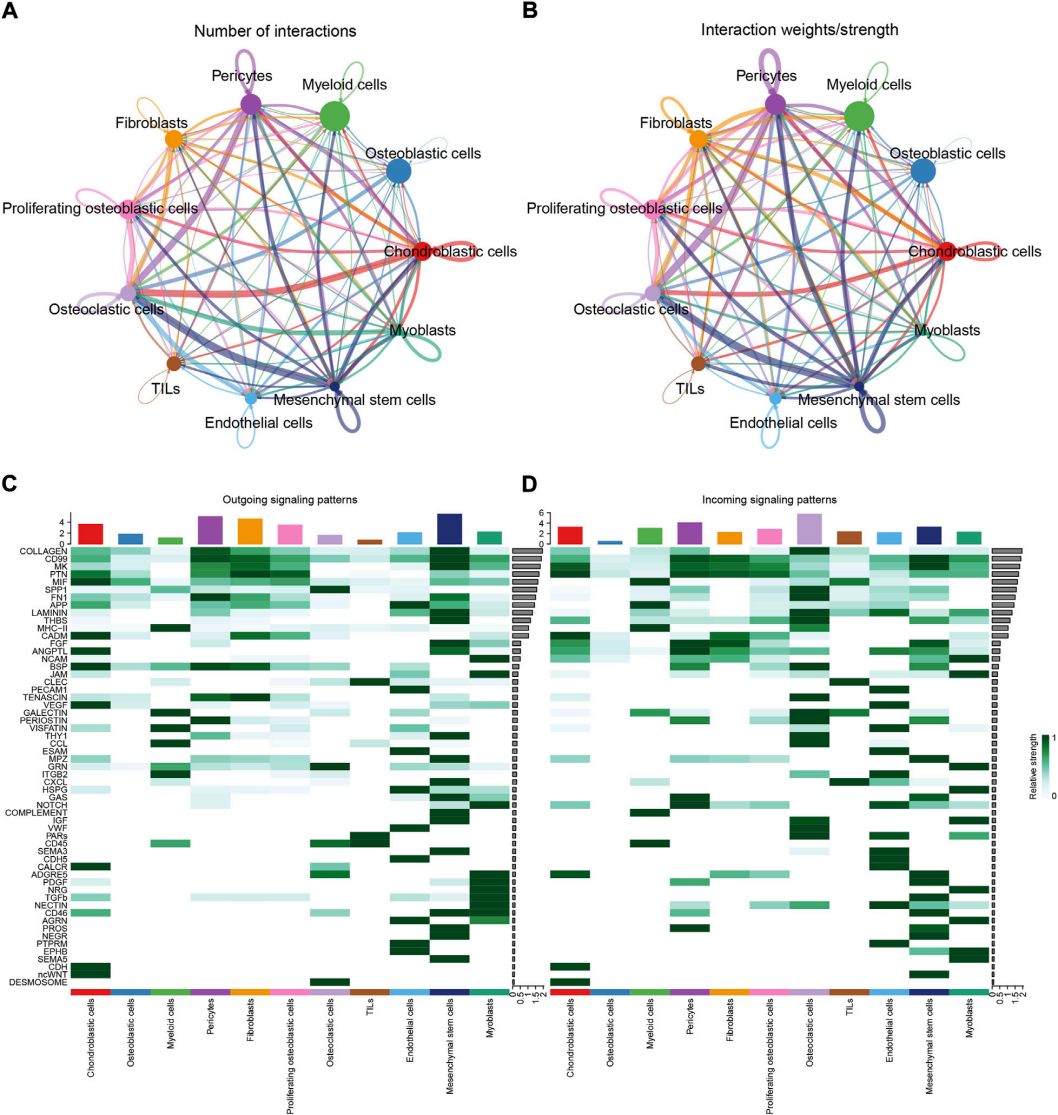
Cell–cell communication network among different cell types. **(A,B)**: the numbers and weights of ligand receptors among all cell types. **(C,D)**: the outgoing and incoming interaction strength among all cell types.

**FIGURE 5:**
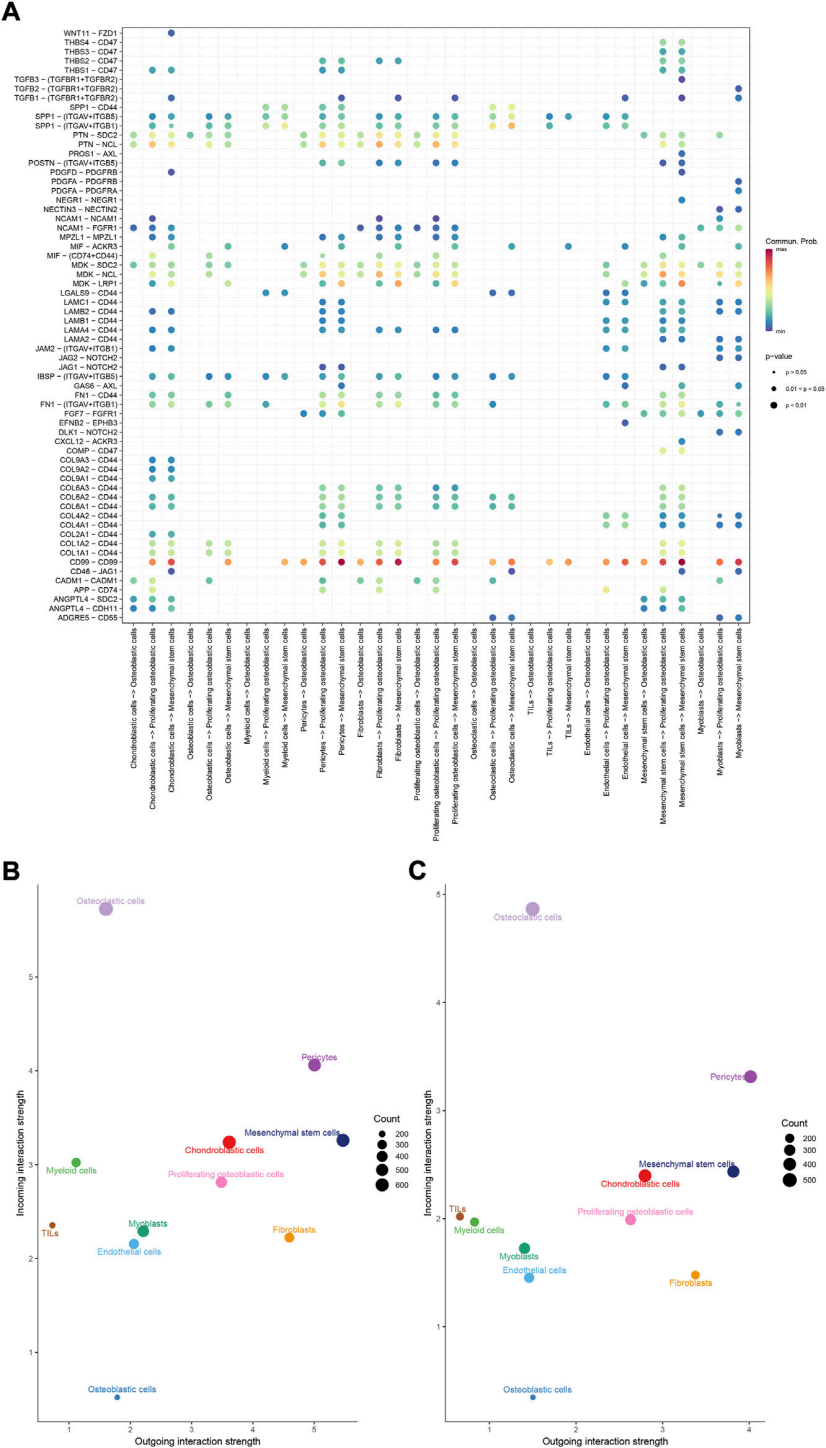
Overview of all ligand-receptor interactions of cells in OS. **(A)**: all ligand-receptor interactions of cells in OS. **(B,C)**: all signaling pathway networks identified, selected signaling pathway networks.

To identify the potential molecular connections between cells, CellChat package (version 1.4.0) of R was utilized to find the potential molecular interactions between ligand-receptor pairings and main cell types in order to build cellular communication networks. First, CellChat was used to analyze cellular communication among the chondroblastic cells, osteoblastic cells, myeloid cells, pericytes, fibroblasts, proliferating osteoblastic cells, osteoclasts, TILs, endothelial cells, mesenchymal stem cells, and myoblasts. The results of the CellChat analysis revealed the numbers and weights of ligand receptors among all cell types (Figures 4A,B). The outgoing and incoming signaling patterns were shown in (Figures 4C,D). The outgoing and incoming interaction strength were shown in (Figures 4B,C) (B: all signaling pathway networks identified; C: selected signaling pathway networks). In addition, all of their ligand-receptor interactions have been identified (Figure 5D).

**FIGURE 6:**
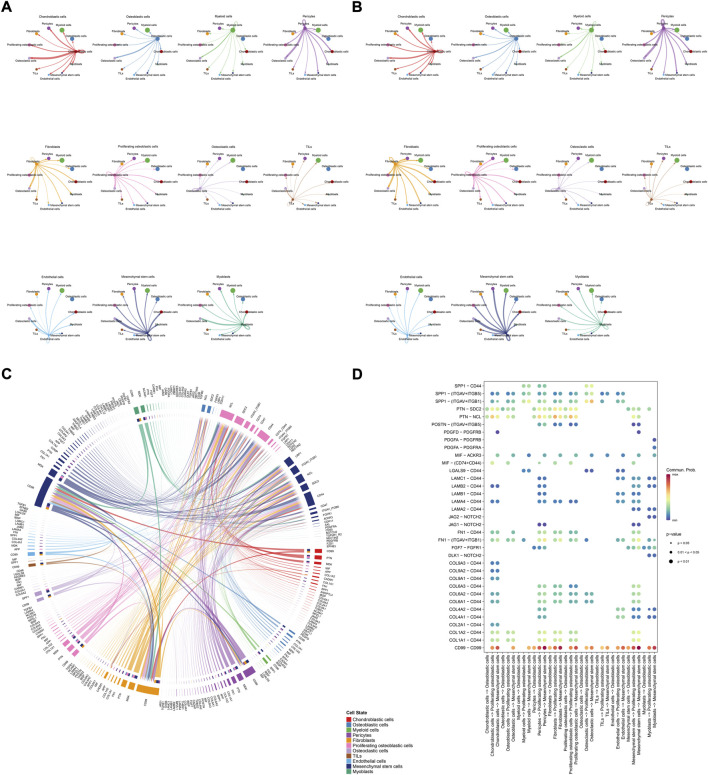
Detailed view of the ligands expressed by each major cell type. **(A)**: a detailed perspective of the ligands expressed by each major cell type and the cells expressing the signal-receiving receptors is provided, the thickness of the lines indicated the numbers of ligand-receptor pairs for each intercellular link. **(B)**: the thickness of the lines indicated the weights of ligand-receptor pairs for each intercellular link. **(C)**: chordal graphs showing potential cellular crosstalk within the OS microenvironment. **(D)**: the selected ligand-receptor interactions related to OS.

**FIGURE 7:**
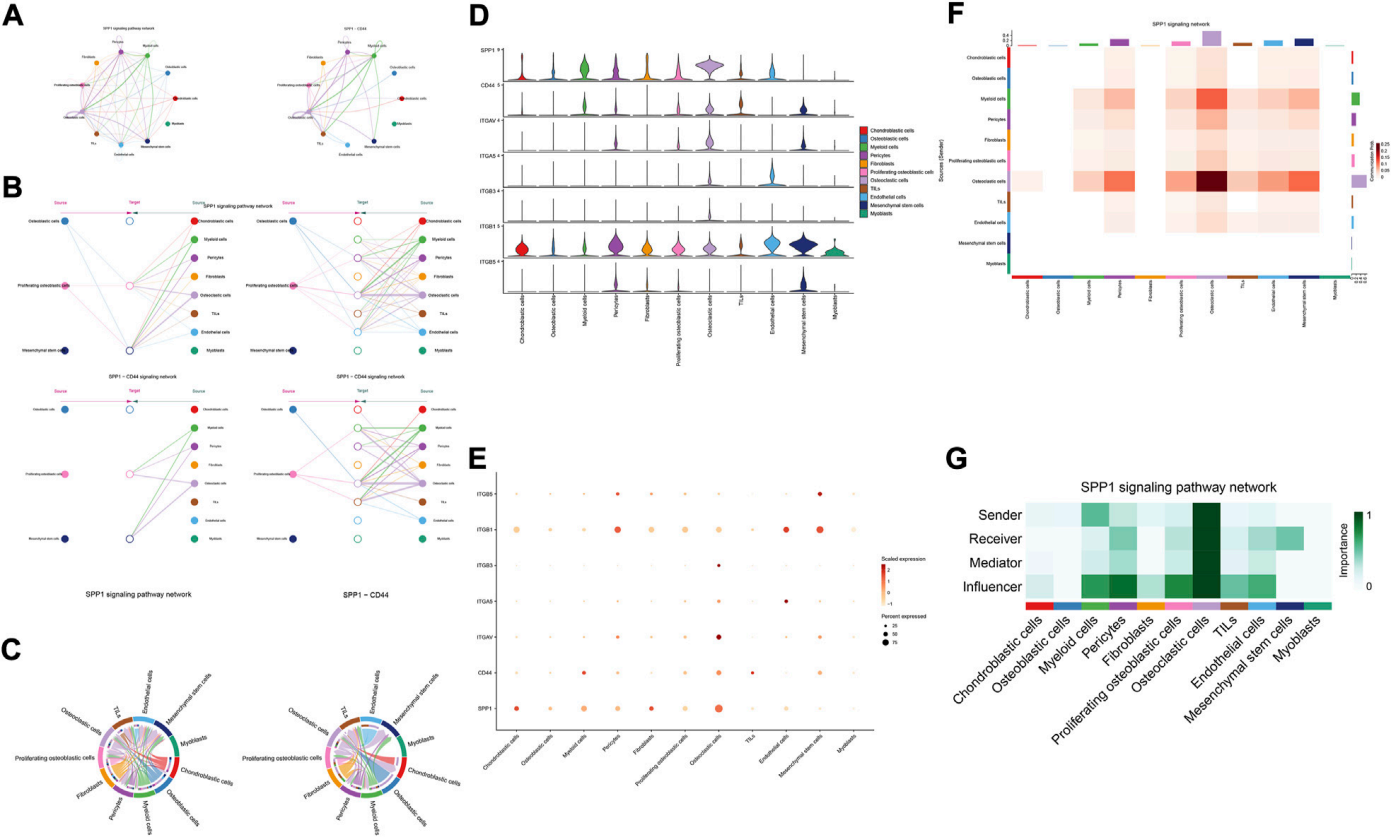
SPP1 signaling pathway. **(A–C)**: ligand-receptor interactions of cells. **(D,E)**: the ligand-receptors expressed by each major cell type. **(F,G)**: the network of SPP1 signaling pathway.

**FIGURE 8:**
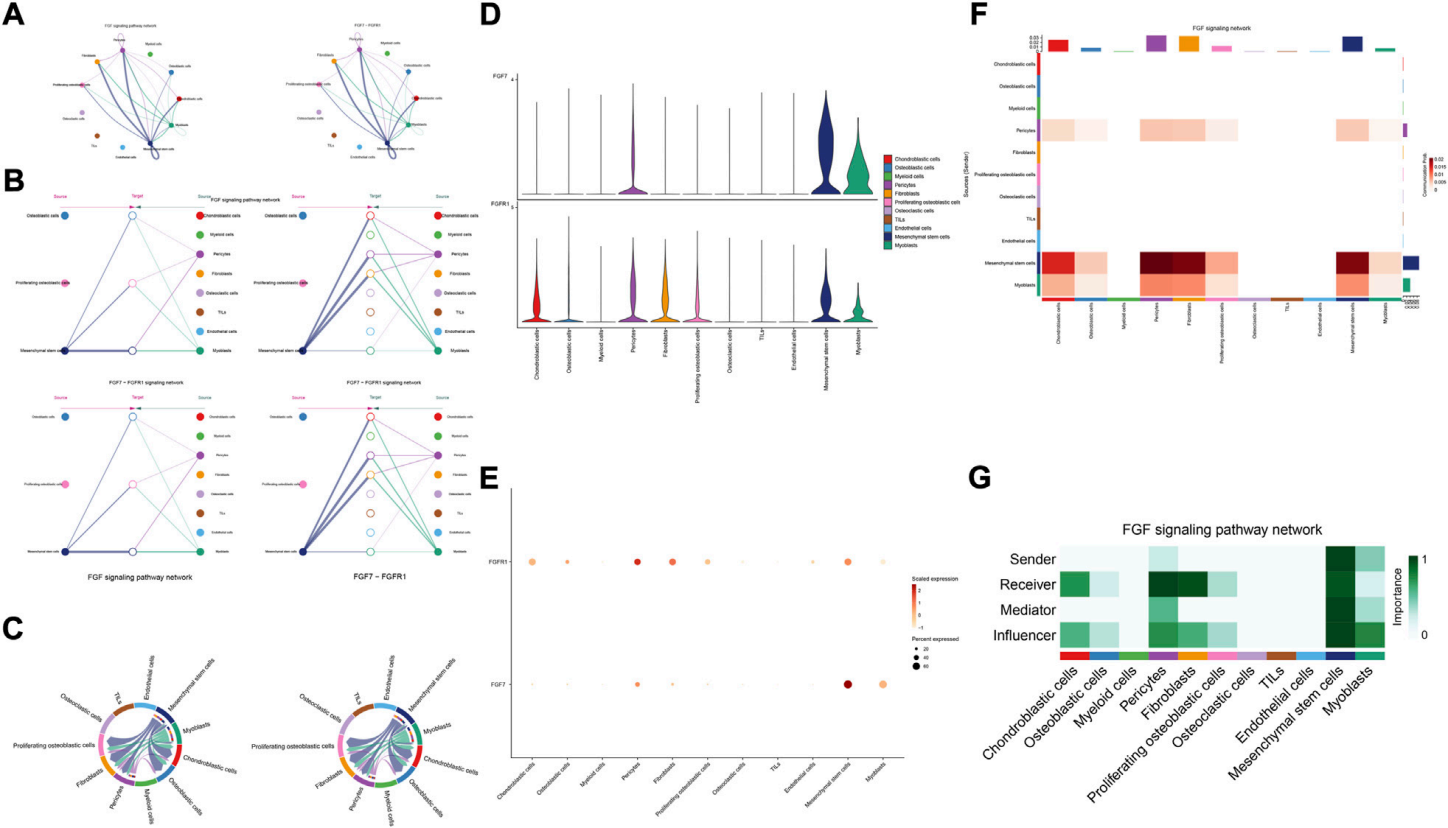
FGF signaling pathway. **(A–C)**: ligand-receptor interactions of cells. **(D,E)**: the ligand-receptors expressed by each major cell type. **(F,G)**: the network of FGF signaling pathway.

**FIGURE 9:**
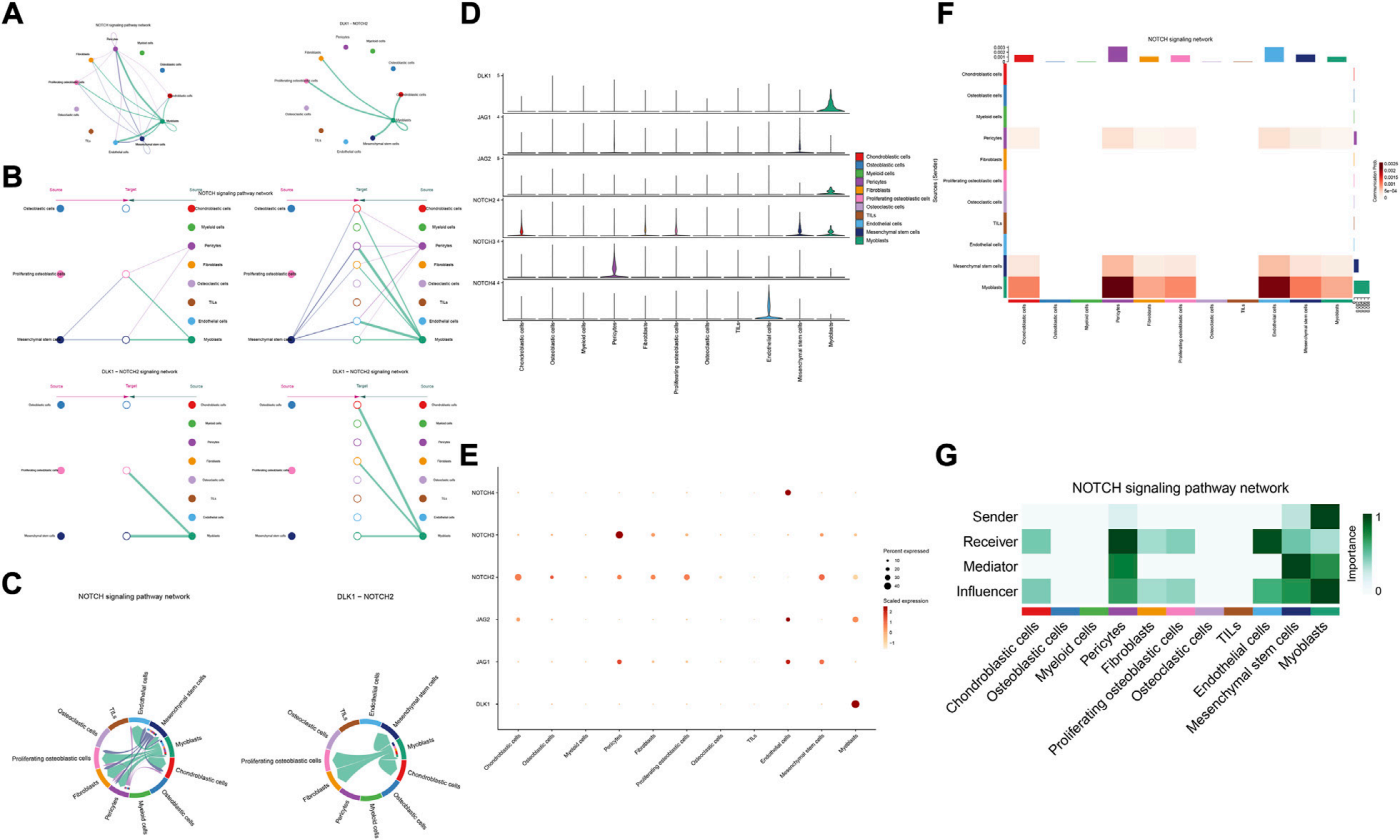
NOTCH signaling pathway. **(A–C)**: ligand-receptor interactions of cells. **(D,E)**: the ligand-receptors expressed by each major cell type. **(F,G)**: the network of NOTCH signaling pathway.

The details of all signaling pathway networks identified were also shown in Figures 6A-C (A: numbers of ligand receptors among all cell types; B: weights of ligand receptors among all cell types; C: chordal graph of ligand-receptor interactions among all cell types). Among the total of 57 signaling pathways, the following signaling pathways were related to osteosarcoma: COLLAGEN (Baumann and Hennet, 2016; Elenjord et al., 2009; Levinson et al., 2002; Yamaguchi et al., 2005), CD99 (Manara et al., 2006; Sciandra et al., 2014; Zucchini et al., 2014), PTN (He et al., 2019; Qin et al., 2022; Sun et al., 2020), MIF(Liu et al., 2014), SPP1(Dalla-Torre et al., 2006; Li et al., 2017), FN1(Saba et al., 2019; Zhou et al., 2019), LAMININ(Heino and Massague, 1989), FGF (Kurogi et al., 1996; Laulederkind et al., 2000; Li et al., 2019; Xu et al., 2010), VEGF (Ji et al., 2020; Lei et al., 2018; Oda et al., 2006; Tsai et al., 2017; Zhang et al., 2019), GALECTIN(Gomez-Brouchet et al., 2010; Miao et al., 2014; Park et al., 2015; Zhou et al., 2014), PERIOSTIN(Ma et al., 2020; Xu et al., 2022), VISFATIN(Cheng et al., 2015; Wang et al., 2019, 2016), ITGB2 (Dai et al., 2018), NOTCH(Jin et al., 2017; Mu et al., 2013; Ongaro et al., 2016; Tanaka et al., 2009; Zhang et al., 2010), IGF (Armakolas et al., 2016; Giatagana et al., 2022; Gvozdenovic et al., 2017; Molina et al., 2019; Tan et al., 2015), VWF(Wang et al., 2020), and PDGF (Chen et al., 2009; Egners et al., 2018; Heldin et al., 1986). The ligand-receptor interactions of these signaling pathways related to osteosarcoma were shown in Figure 6D. Furthermore, according to the results of this research, the potential communication of mesenchymal stem cells, proliferating osteoblastic cells, and osteoblastic cells mainly revolved around SPP1 (Figure 7), FGF (Figure 8), NOTCH (Figure 9).

### Differentiation trajectory analysis of mesenchymal stem cells, proliferating osteoblastic cells, and osteoblastic cells

**FIGURE 10:**
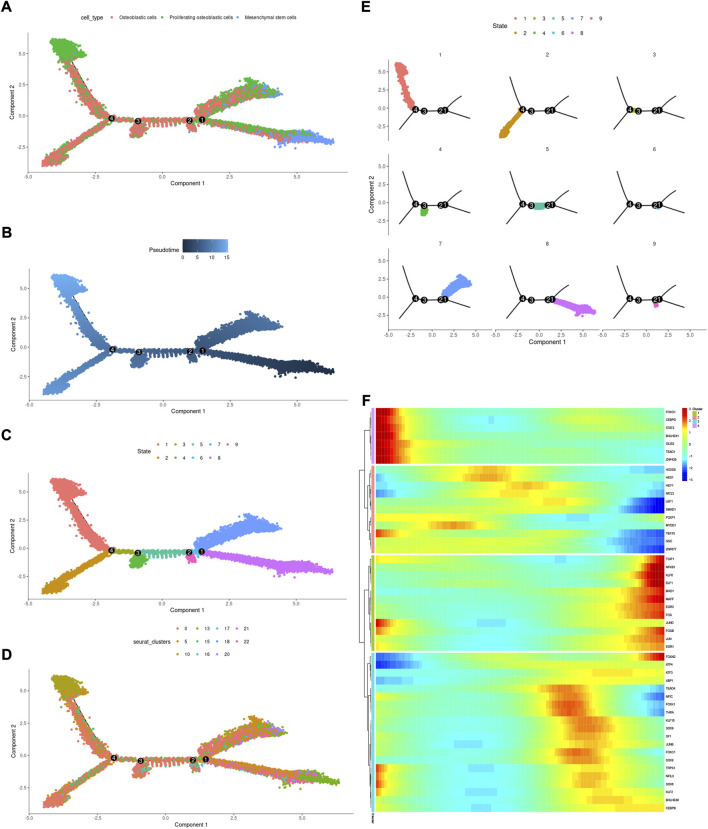
Trajectory analysis of mesenchymal stem cells, proliferating osteoblastic cells, and osteoblastic cells. **(A–E)**: trajectory plots showing the differentiation of these cell types. **(F)**: heatmap displaying the scaled expression of dynamic genes across time. The rows of the heatmap reflect genes exhibiting dynamic changes along the pseudotime, and these genes have been grouped into four categories based on their expression pattern over the pseudotime.

Cell state transmission was evaluated using pseudotime analysis based on the Monocle package. The mesenchymal stem cells, proliferating osteoblastic cells, and osteoblastic cells were subjected to differentiation trajectory analysis. We performed pseudotime analysis to explore the cell-state transitions among mesenchymal stem cells, proliferating osteoblastic cells, and osteoblastic cells (Figures 10A–E). Furthermore, we plotted the heatmap of the differentiation trajectory among these cells (Figure 10F). The results of trajectory analysis revealed that osteoblastic cells followed a differentiation trajectory that primarily began with clusters of mesenchymal stem cells and proliferating osteoblastic cells, from which they differentiated into osteoblastic cells.

### Single-cell regulatory network of mesenchymal stem cells, proliferating osteoblastic cells, and osteoblastic cells

**FIGURE 11:**
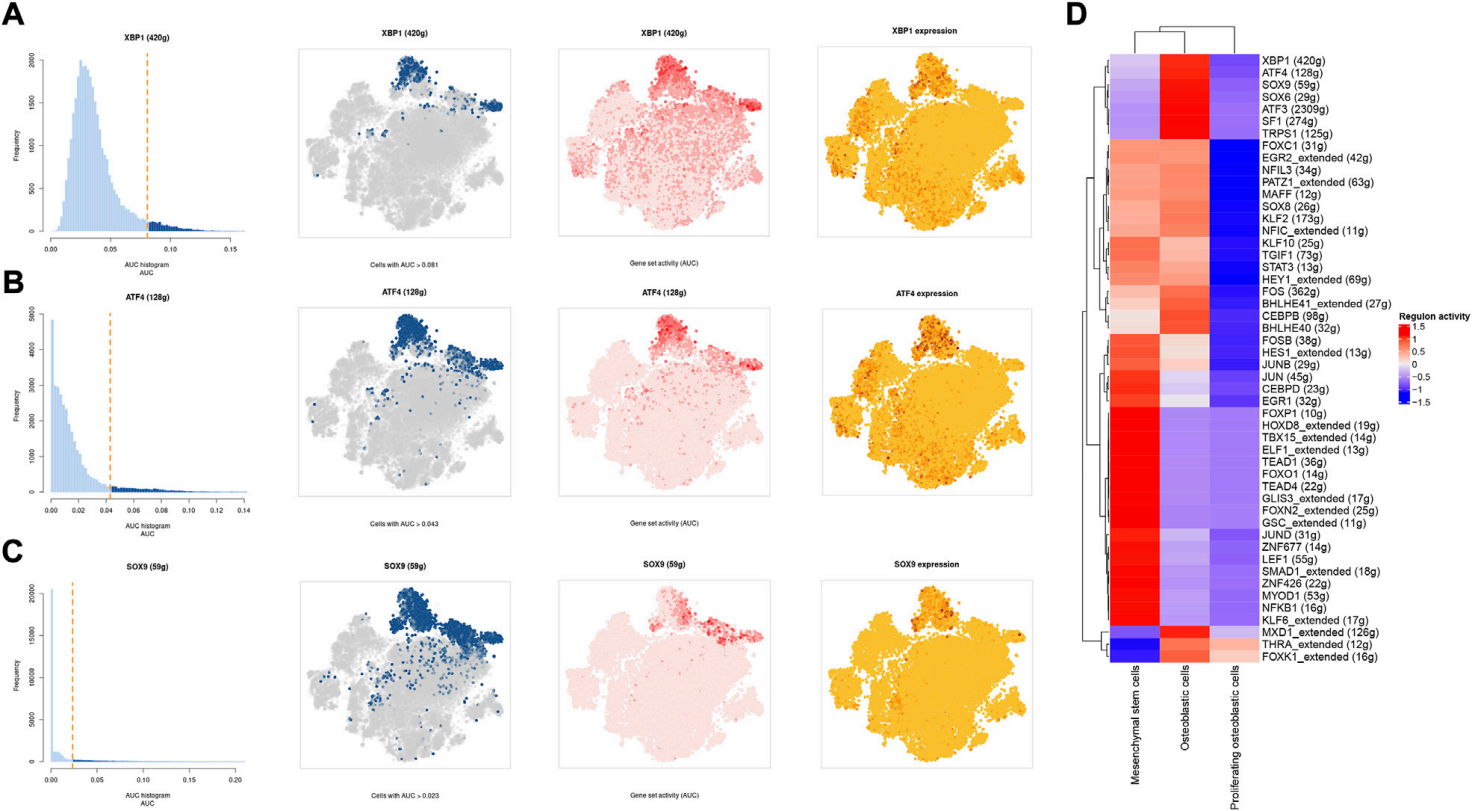
The SCENIC analysis predicted the TF. **(A–C)**: tSNE plots and histograms show the top three TF activities in mesenchymal stem cells, proliferating osteoblastic cells, and osteoblastic cells. **(D)**: Heatmap showing the top 50 TF in these cell types.

A SCENIC analysis was conducted to detect the TFs of mesenchymal stem cells, proliferating osteoblastic cells, and osteoblastic cells. The genes of TFs (XBP1(Yang et al., 2015; Yu et al., 2022), ATF4 (Luo et al., 2017, 2019; Xian et al., 2017), and SOX9(Y. Chen S. et al., 2020; He et al., 2017; Wang et al., 2018)) were significantly activated in osteoblastic cells (Figures 11A–D), and were demonstrated to be expressed in osteosarcoma.

## Discussion

Osteosarcoma is the most common malignant bone tumor in children, teens, and young adults with a median age of 16 years. It accounts for approximately 56% of bone sarcomas, and metastasis is the primary reason why treatment fails and the prognosis is poor (Chen et al., 2021). Despite previous molecular biology investigations having offered considerable information on the pathogenesis of osteosarcoma, the mechanisms that regulate its several oncogenic insults necessary for osteosarcoma start and development remain unknown (Isakoff et al., 2015; Kansara et al., 2014). It remains a serious concern due to poorly characterized carcinogenesis processes and restricted therapeutic options. So, it is essential to find important subpopulation driver mutations that promote diversity, expansion, invasion, and eventual colonization of other areas of the body. In addition, the potential cellular communication networks in osteosarcoma and the influence of tumor heterogeneity on cell aggregation are crucial.

Single-cell RNA sequencing (scRNA-seq) can show variation within cell populations. It could discriminate tumor cells from non-tumor cells and examine intercellular connections within the tumor microenvironment by analyzing transcripts inside individual cells. It is helpful to find unique cell types, look into tumor heterogeneity and potential networks of cell-to-cell communication, and show different developmental paths. This can give a theoretical foundation for future research into the molecular processes of OS growth and metastasis (Guo et al., 2022).

Mounting clinical and experimental data suggests that osteosarcoma stem cells, which originate from mesenchymal stem cells, may be the biological genesis of osteosarcomas and demonstrate osteoblastic differentiation, producing malignant osteoid (Brown et al., 2017; Xi et al., 2000). In addition, osteosarcoma is strongly connected with the osteoblastic lineage and displays osteogenic differentiation-related activities in proliferation, extracellular matrix secretion, and induction of ossification (Zeng et al., 2022). So, in this study, potential cellular communication networks among mesenchymal stem cells, proliferating osteoblastic cells, and osteoblastic cells were identified through comprehensive analysis of osteosarcoma single-cell RNA sequencing (scRNA-seq), illustrating the complex regulatory network in the advanced osteosarcoma microenvironment. Moreover, we performed transcription factor regulatory network analysis and trajectory analysis on these cells.

The results of cellular communication networks showed that mesenchymal stem cells, proliferating osteoblastic cells, and osteoblastic cells are mainly involved in SPP1, FGF, and NOTCH signaling pathways. The SPP1 gene (osteopontin, secreted phosphoprotein 1) encodes a protein with several activities, including bone remodeling, adhesion, tumor invasion, and metastasis (Dalla-Torre et al., 2006). It is generated by a variety of cell types, including osteoblasts, osteoclasts, and endothelial cells (Liu et al., 2013; Wang and Yang, 2015). SPP1 is now of interest in carcinogenesis, Lysosomal-associated membrane protein 3 (LAMP3) enhances osteosarcoma cell invasion via SPP1 signaling (Li et al., 2017). In colorectal cancer (CRC), SPP1 was highly upregulated and increased CRC metastasis by promoting epithelial-mesenchymal transition (EMT) (Xu et al., 2017). In addition, previous research found inhibition of the SPP1 gene may have therapeutic benefits for tongue cancer and may be a useful target for therapy (Zhang et al., 2020). Moreover, in pancreatic tumor microenvironment factors, the SPP1-CD44 axis can promote cancer stemness (Nallasamy et al., 2021). In head and neck squamous cell carcinoma (HNSCC), SPP1 overexpression is prognostic of worse survival results (Bie and Zhang, 2021). However, some scholars found that overexpression of SPP1 was correlated with improved overall survival, event-free survival, and relapse-free survival at diagnosis in osteosarcoma (Dalla-Torre et al., 2006). The results of our study revealed that through the SPP1-CD44 signaling pathway, myeloid cells, pericytes, and osteoclast cells can impact on mesenchymal stem cells and proliferating osteoblastic cells. Moreover, in these cellular communication networks, osteoclasts play a role as major senders, mediators, and influencers of the signal.

Fibroblast growth factor (FGF) signaling is essential for embryonic organ development and the progression of tumors (Brewer et al., 2016) and increases proliferation, invasion, and epithelial-to-mesenchymal transformation of tumor cells. (Bono et al., 2013). In the majority of malignancies, numerous FGFs are increased, and different FGF receptor (FGFR) subtypes are activated on tumor and stromal cells. (Turner and Grose, 2010). In addition, cancer, inflammation, and the resistance of tumor vascularization to VEGF inhibitor therapy have all been linked to FGFR signaling. (Beenken and Mohammadi, 2009; Casanovas et al., 2005; Fischer et al., 2007; Turner and Grose, 2010). Moreover, in the development of cancer, pathological FGF/FGFR signaling enhances cross-talk between oncogenic cells and its microenvironment, ultimately causing cancer cell proliferation, angiogenesis, and migration. (Li et al., 2018). For example, in the tumor microenvironment of esophageal cancer, NCAM- and FGF-2-mediated FGFR1 signaling modulates the survival and migration of tumor-associated macrophages and cancer cells (Takase et al., 2016). Additionally, FGFs activate myeloid cells, macrophages linked with tumors, cancer-related fibroblasts, and osteoclasts (Berardi et al., 1995; Collin-Osdoby et al., 2002; Itoh, 2007). Recent studies have found that in the evolution of osteosarcoma, FGF has emerged as a crucial regulator. According to previous research, LHX9 is critical for the proliferation, migration, invasion, and metastasis of OS cells via the FGF and TGF−/−catenin signaling pathways (Li et al., 2019). Some scholars have found that through the FRS2/TGF−/−catenin pathway, FGF-induced LHX9 controls osteosarcoma development and migration (Li et al., 2019).

Our research found, through the FGF7-FGFR1 signaling pathway, mesenchymal stem cells, pericytes, and myoblasts may influence mesenchymal stem cells, proliferating osteoblastic cells, and osteoblastic cells. High quantities of FGFR1 and FGF7 were detected in mesenchymal stem cells and pericytes. In addition, in these cellular communication networks, mesenchymal stem cells and pericytes serve as important signal senders, mediators, and influencers.

The Notch pathway regulates various mechanisms that control morphogenesis, lineage determination, apoptosis, and proliferation in some malignancies (Bray, 2006), and has been identified as both a tumor suppressor and an oncogene (Jin et al., 2017; Tanaka et al., 2009; Zhang et al., 2010). The Delta-Serrate-Lag (DSL) family of ligands (jagged 1/Jag1, Jag2, delta-like-1/DLL1, DLL3, and DLL4) on the surface of a cell connect with a membrane-bound Notch receptor (Notch1-4) on a different cell to start the Notch signaling pathway, a crucial step in normal bone growth that is also implicated as a critical mediator in a variety of different malignancies (Iso et al., 2003).

According to previous research, the notch pathway is strongly related to the development of osteosarcoma. Erk phosphorylation promotes osteosarcoma proliferation and migration in response to Notch stimulation (Qin et al., 2019). By activating cell division cycle 20, Notch-1 increases the evolution of osteosarcoma to a malignant state (Gao et al., 2020). The elevated expression of Jagged1 is intimately associated with osteosarcoma metastasis and recurrence. On the contrary, the knockdown of Jagged1 significantly reduced osteosarcoma cell proliferation, migration, and invasion (Zhang et al., 2021). Additionally, Notch signaling also regulates the immune system of the tumor microenvironment. Inhibiting the Notch signaling system enhances the polarization of TAM towards the M2 genotype, which in turn promotes the growth and spread of osteosarcoma (Ren et al., 2020). Our research found, through the DLK1- NOTCH2 signaling pathway, myoblasts may influence mesenchymal stem cells, proliferating osteoblastic cells. Additionally, myoblasts serve as important signal senders, mediators, and influencers.

One of the most prevalent issues in the development of human cancer is the dysregulation of transcription factors, which plays a role in the pathogenesis of the disease. The SCENIC analysis revealed that the regulon activity of XBP1, ATF4, and SOX9 were down-regulation in both mesenchymal stem cells and proliferating osteoblastic cells. X-box binding protein (XBP1) is a significant transcriptional regulator of the unfolded protein response. Lack of oxygen stimulated the transcription and translation of XBP1 mRNA, resulting in an increase in the activity of XBP1 protein (Romero-Ramirez et al., 2004). It was initially identified as a crucial regulator of major histocompatibility complex class II (MHC) gene expression in B cells (S. Chen Y. et al., 2020). High XBP1 levels were associated with advanced clinical stages, a high malignancy index, and a poor tumor necrosis rate in OS. XBP1 knockdown decreased OS cell growth and survival in culture (Yang et al., 2015). Recent studies have shown that XBP1 increases the susceptibility of HOS osteosarcoma cells to pyropheophorbide- α methyl ester-mediated photodynamic remedies (Yu et al., 2022). Activating transcription factor 4 (ATF4), a major regulator of the integrated stress response system, activates transcription of a group of transcriptional silencing genes that regulate cell survival and death (Ishizawa et al., 2016). In recent years, numerous investigations on the involvement of ATF4 in osteosarcoma have been reported. In human osteosarcoma, suppression of GRP78 increases ATF4-induced cell death via deubiquitination and stability of CHOP(Luo et al., 2017). Moreover, through endoplasmic reticulum (ER) stress-mediated PERK/eIF2/ATF4/CHOP activation and Wnt/β-catenin signal suppresses the development of human osteosarcoma (Zhao et al., 2020). ATF4 devastates RET by inhibiting nonclassical GRP78 to increase osteosarcoma chemosensitivity to bortezomib (Luo et al., 2019). Sex-determining region Y (SRY)- box 9 protein (SOX9) is a crucial transcription factor in a variety of illnesses, particularly in malignancies. Recent research has revealed that SOX9 plays an important function in the control of the tumor microenvironment (TME). Furthermore, SOX9 signaling or SOX9 controlled signaling pathways play an important role in cancer development and metastasis (Panda et al., 2021). Additionally, by means of a Sox9-Mediated positive feedback loop, MAFB contributes towards the progression of cancer stemness and tumorogenesis in osteosarcoma (Y. Chen S. et al., 2020). Moreover, previous study has found the cFOS-SOX9 axis of chondroblastic osteosarcoma reprograms bone marrow derived mesenchymal stem cells into chondroblastic cells (He et al., 2017).

In conclusion, this study uncovered the potential cellular communication networks between several cell types in advanced osteosarcoma. The SPP1, FGF, and NOTCH signaling pathways may play a crucial role in osteosarcoma TME regulation. This research may bring fresh insights into the pathophysiology of osteosarcoma’s molecular processes. However, this paper has the following limitations: no additional experiments were conducted to validate the data mining findings presented in this study; no further validation using the bulk RNA-seq database of osteosarcoma.

## Data Availability

The datasets presented in this study can be found in online repositories. The names of the repository/repositories and accession number(s) can be found in the article/Supplementary Material.
